# George Frankl: an undervalued voice in the history of autism

**DOI:** 10.1007/s00787-020-01622-4

**Published:** 2020-08-27

**Authors:** Filippo Muratori, Sara Calderoni, Valeria Bizzari

**Affiliations:** 1 Department of Developmental Neuroscience, IRCCS Fondazione Stella Maris, Pisa, Italy; 2 Department of Clinical and Experimental Medicine, University of Pisa, Pisa, Italy; 3 Center of Psychosocial Medicine, Department of Psychiatry, Clinic University of Heidelberg, Heidelberg, Germany

**Keywords:** George Frankl, Word language, Affective language, Affective contact, Asperger, Kanner

## Abstract

This paper aims to propose that the psychiatrist George Frankl had more than a marginal role in the early history of autism. Frankl’s conception of autism as characterized by a lack of affective language has influenced both Asperger and Kanner. First, this proposal is historically supported; second it is corroborated by Frankl’s unpublished manuscript on Autism. We found that Frankl’s perspective about autism was, and still can be, considered innovative for multiple reasons. Specifically, Frankl proposed that autism could cover a spectrum of conditions; that it is a state of mind that is not necessarily abnormal; and that it is a neurobiological condition, which primarily needs to be understood by others. Finally, Frankl’s concepts of affective contact and affective language are reconsidered with reference to contemporary neuropsychology from which autism emerges not as a higher-order cognitive deficit, but as a result of an impairment of primordial ability to process low level sensory, motor and perceptual information gained through experiencing other persons.

## Introduction

For almost 70 years, the origins of autism as a distinct diagnostic category have been linked with two names: Hans Asperger, a pediatrician who worked in Wien and Leo Kanner, a psychiatrist who worked in Baltimore, Maryland.

It is usually reported that the concurrent choice to call a new psychiatric disorder of children ‘autism’ by both Asperger and Kanner was a strange coincidence (‘one of the great coincidences of the twentieth century medicine’, as it was written by Silberman [36]), as the two authors did not know each other. For example, in his recent comprehensive book on joint attention, Peter Mundy states that “Leo Kanner and Hans Asperger, respectively and independently, discovered evidence of the distinct syndrome of what is now called autism spectrum disorder” [27].

In this brief report, we refute this recurrent position trying to bring evidence to the pioneering role that George Frankl played in identifying the new psychiatric syndrome. In doing so, we must first recognize our debt to several authors: Silberman [36], who is credited with introducing Frankl to the history of autism; Robison [34], who has highlighted the important role that Frankl would play on ‘both sides of the Atlantic’; and to Todd [40], whose intensive dissertation has dug up the nature of the relationship between Kanner and Frankl after his arrival in Baltimore. Silberman, Robison and Todd have all said their piece and ‘left the door ajar’. Here, we step into the breach. In particular, we would like to focus on the fact that historical circumstances led Kanner and Asperger to become famous, while George Frankl and his ideas were unfortunately forgotten. This paper has the main aim of retracing the early history of autistic diagnosis and of delineating the role played by George Frankl within this framework.

## Method

Our survey on the history of the autistic diagnosis can be divided into two main parts. First, we focused on papers and books about the history of autism, using a cross-reference analysis (with the words: autism, Asperger, Kanner, diagnosis) in the MEDLINE (PubMed) database and Google search engine. In doing this, we identified a huge gap due to the absence of George Frankl in the literature, with the exception of three works [34, 35, 40].^1^ Within the framework of the history of the autistic diagnosis, the contributions of Kanner and Asperger are very well recognized, while there is no space for George Frankl’s role. In response, we decided to shift the focus to the possible contribution of George Frankl in the early history of autism.

In the second (and more consistent) part of our inquiry, we started to look for more information about Frankl’s life and work. We looked back at his biography (using the Archive of the Deutsche Gesellschaft für Kinder und JugenMedizin, where it is possible to find a brief biography), and we wrote to the University of Kansas that kindly gave us the contacts to reach the Library of Kansas (https://spencer.lib.ku.edu), where Frankl’s [16], entitled *Autism and Childhood. An Attempt of Analysis*, is kept. This document was kindly provided to us; we then translated and studied it. The Italian translation was published in 2019 by the publishing house Fioriti Editore in Rome [28]. We then focused on Frankl’s two other papers (found online: [17, 18]). We searched for similarity and theoretical continuities among the three works, and we also compared these papers to Kanner and Asperger’s autistic descriptions. The cross-checking of the historical data with the theoretical observations allowed us to hypothesize the role of George Frankl within the early development of autistic diagnosis.

## George Frankl has influenced both Kanner and Asperger

In 1931, at the beginning of his career after his medical degree, the young medical doctor, Hans Asperger (1906–1980), was introduced at the Heilpädagogische Station (created in 1911 by Erwin Lazar, 1877–1932, and subsequently known as Lazar Clinic) by his mentor Franz Hamburger who was an active member of the Nazi party. There, he began to work with the senior psychiatrist, Georg Frankl (1897–1975, the first name changed to George after arriving in the USA), who was the chief diagnostician at the Lazar Clinic and with the psychologist Anni Weiss (1897–1991). Asperger was a postdoc when both Frankl and Weiss were senior clinicians—already were working on cases similar to those later described as autistic [17, 43]. In 1935, Joseph Michaels [26] published on the *American Journal of Orthopsychiatry* a report of his visit to the Lazar Clinic. In this text, Hans Asperger, who was already appointed head of the ward still without the habilitation, was not mentioned, whereas the only two people mentioned in relation to the work on ‘autistic children’ were Anni Weiss and George Frankl. The latter is described as one who believed this was due to the youth’s poor understanding of the emotional content of the spoken word; concurrently, Weiss is described as focused on hidden intelligence, fixations and communication impairments.

Vienna in the 1930s was the capital city of an oppressive, authoritarian political system and although Jews had lived there for over a 1000 years, anti-semitism was prolific. This political climate forced Weiss, in 1934, and then Frankl, in 1937, to leave Vienna, even before the Anschluss of March 1938, because they were both Jewish. Then, their works were left uncited and forgotten. A similar story played out with the most well-known Russian psychiatrist Grunya Sukhareva who had published a paper on children with schizoid personality disorders in 1926. Her description was very similar to that made by Asperger 20 years later. When Wolff translated Sukhareva’s paper in English [44], she considered the possibility that Asperger read it as originally written in German, and chose not to quote it, or he may not have been permitted to credit Sukhareva because she was Jewish.^2^ As a consequence, the initial, deeper understanding of autism was informed by writing of professional women, but their role was not acknowledged, likely as a result of the academic culture of the times.

Frankl fled to Maryland where his future wife Anni Weiss was waiting for him. Leo Kanner, another Jewish MD, escaped from Europe many years before and managed to procure entry visas in the USA to many Jewish doctors persecuted by the Nazis; among them was George Frankl. In 1938, Frankl began to work with Kanner at the Johns Hopkins Hospital. Frankl’s merits and acumen were immediately recognized by Kanner, who wrote to Bernard Sachs: ‘I have become very much interested in what Dr. Frankl calls the affective contact of children…in that it opens a new approach to the observation and understanding of the mental life of the child’ (Kanner to Sachs, quoted by Todd [40, p. 253]). Again in 1943, 3 years after Frankl left for Texas, he wrote to the publisher of *The Nervous Child:* “The more I read Frankl’s paper, the more I am impressed by it and the more I realize what a gem it is. My own paper on autistic disturbances of affective contact is now just taking shape…I plan to have his paper precede mine” (Kanner’s personal communication, quoted by Robison [34, p. 6]).

Nevertheless, the final decision of the editor was to publish Frankl’s paper [18] after that of Kanner [24], and even if Kanner quoted in his paper Frankl as the observer of Donald Triplett—the most famous of his 11 cases—Frankl’s contribution was lost. Kanner visited Donald Triplett in 1935, well before the arrival of Frankl in Baltimore, but he did not detect the autistic features in that child; only after the arrival of Frankl, and his detailed child observation and description, Kanner began to recognize Donald T. as a prototypic case of autism. It can be assumed that before meeting George Frankl, Kanner missed a frame of reference in which to place what he was observing in some of his young patients (see also [14]). Kanner found the theoretical and clinical framework he was looking for in Frankl’s concept of affective contact. It is worth emphasizing that the title of the famous 1943 paper was ‘Autistic disturbances of affective contact’ and not simply ‘Autistic children’ as in the follow-up paper [25]. Throughout the entire paper, Kanner seems to be inspired by Frankl: he comments on patients’ use of language and words, and he states that: “None of these remarks was meant to have *communicative* value. There was no affective tie to people” [24, p. 227–228]; “During the interview there was no kind of affective contact” [24, p. 229]; “He never used language as a means of *communicating with* people” [24, p. 237]. The emphasis given to the role of communication, and to its value within the syndrome is the same we can find in Frankl’s [16], where he asserts that communication means contact with people; contact is a matter of affect. Kanner adds other pathognomonic features (e.g. autistic loneliness, excellent rote memory, echolalia, obsessive desire for sameness), but what is striking here is the emphasis put by Kanner on the notions of ‘affect’ and ‘communication’, which are at the very core of the disorder for Frankl’s conception.

When Frankl escaped to the USA, Asperger^3^ had been the head of the ward for 2 years. In this framework, it is difficult to hypothesize what Asperger thought of his senior diagnostician George Frankl and how much he was influenced by his thinking on affective language. In fact, Asperger never quoted Frankl in his papers. However, some links may be detected. For example, in 1938, the year of the Anschluss, Asperger gave a lecture under the tutelage of Franz Hamburger: “The mentally abnormal child” (now traduced by Falk [15]). Here [2], he first described how children are educated: “…they have normal relationship, not because they understand the content of the educator’s instructions, but because they instinctively understand the educator’s tone of voice, facial expressions, and gestures”; then he focused on difficulties in education of those whom he defines as ‘autistic psychopaths’: “It is just this instinctive understanding that is severely disturbed in our children. All of their abnormal symptoms can be deduced from this disturbance: the lack of instinctive comprehension that accounts for the failure to respect authority and the ongoing failure to understand discipline.”

It is possible to discover Frankl ideas on disturbances of affective language comprehension that he had expressed very vividly in ‘Ordering and Obeying’ [17]. Frankl wrote:

I remember the scene between a small child and his mother. He was a 5-year-old boy, particularly restless and rowdy; the angry mother muttered behind him, in a monotonous voice and a face without expression…the child barely perceived this weak litany, and he did not care at all to obey. It is an example of how giving orders can be inadequate. Only when we observe the gestures and facial expressions that accompany giving orders does this become clear to the viewer and to those who receive it. It is obvious that this is not only valid in giving orders, but more broadly in human relationships when it comes to communicating. If educational actions are not integrated with affective language, they do not find the right emotional contact with children, and then, these educational actions have a strangely empty effect and can be confusing for the child and also for the spectator (Frankl [17, p. 464], our translation).

After this lecture, Asperger was focused on preparing his thesis that was edited in 1944 (subsequently translated in English by Uta Frith in 1991). Also in this work, it is possible to find Frankl’s influences:

Long before the child understands the words of the educator, he learns to obey not abstract words, but to look, to the tone of the voice, to the expression of the face, to gestures … what in the first place causes a child to obey is not the effect of the content of words but the emotional state of the educator that shines through his words … even the infant, even the stranger, and even the dog while they do not understand the meaning of the words, understand the affectivity that emanates from them. (Asperger, transl. [3, pp. 46–47])

In these passages, ideas on affective language are reported several years after their first description by Frankl. Still in 1977, Asperger states: “If my attention had not been attracted to the bodily signs of affective states, I would never have been able to discern the autistic personality” (Asperger [4], quoted in Todd [40, p. 236]). So, after many years, Asperger seems to acknowledge its debt to the one who initiated him, in the years of Nazism, into the importance of affective language but refrained from any explicit recognition.

## Three main topics in Frankl’s writings

### Affective language and affective contact

Robison describes Frankl, between Asperger and Kanner, as a hero who first observed the autistic disconnection between facial expression, body language, and speech. This topic is widely developed by Frankl in the unpublished manuscript: *Autism in Childhood: An Attempt of Analysis*, held by University of Kansas’s Library, one of the latest places where Frankl, together with his wife Anni Weiss, worked after the 2 years in Baltimore with Leo Kanner. The manuscript is an unfinished work of which only the first chapter remains; it is composed of 62 sheets, still with pen corrections, waiting to be concluded and published. The exact date is also unclear, although in some rare citations it is quoted as a thesis written in 1957; but Frankl would have been over 60 years old at that time, and it is unlikely that he was still struggling with a thesis. Rather, this text seems to be an attempt, developed by Frankl over his years in Kansas, to organize his ideas on autism as a disorder of affective language, while in those years autism was known as ‘Kanner disease’, Asperger’s thesis yet to be translated.^4^

Frankl’s perspective on autism remained unexplored, but we think that it is worthy of attention. Frankl offered an analysis of autistic *language*, and his survey was guided by the question: How does the autistic child communicate or not communicate with the people around him?

This perspective is deeply developed in Frankl’s [16] that explores the hypothesis that the state of autism complements the state of “being in communication with people”. A person is either in one condition or in the other. Starting from the assumption that talking is different from communicating, Frankl distinguishes between the word language and the affective language. While the *word language* involves all verbal communicative symbolizations, the *affective language* matches non-verbal communicative symbolizations (e.g. facial expression, body gestures, the modulation of articulate and inarticulate sounds) and, in his view, it comprises true communicative symbols, which have validity in the subject’s family, country, and to some extent, worldwide. It is a method of communication, beyond the boundaries of the spoken language that the baby learns early on. The difference between ‘affective’ and ‘word’ language proposed by Frankl in his papers from 1934 to 1957 recalls what the philosopher Thomas Reid identified as the difference between ‘natural’ (affective) and ‘artificial’ (word) language. Reid wrote: “…it is by natural signs that we give force and energy to language, and the less language has of them, it is the less expressive and persuasive” [30]. Everyday language is always a fusion and integration of word language and affective language from which it derives a ‘good contact with persons’. According to Frankl, the ‘good contact’ comprises: (1) a physical component and (2) an intentionally communicative, symbolizing representation. In order to account for this dual characterization, Frankl makes the example of rage: usually rage has its own bodily features that express aggression *toward somebody* (I am angry *at you*; I want to hurt *you*; in a fit of anger I can scowl *at somebody*; I can shake my fist *at him* or punch *him*). In other words, the opponent, the object of my rage, is an essential part of the rage itself. This e*xpressive and intentional directness* is missing in autism; its very core seems to be exactly the inability to tune into the world.

The centrality of the affective language disturbances is very apparent in high functioning conditions, where not all the intersubjective, communicative layers are impaired (indeed, they can maintain a “speaking relationship with people although their *contact with* them is interrupted”—Frankl [16, p. 53]). In low-functioning or in nonverbal children (‘autistic mutism’ in Frankl manuscript), this break between affective and word language (already developed in the 1934 paper, as previously mentioned) could be less explicit. Frankl is very careful not to confuse an autistic child who does not speak or who is only echolalic with a child who is not communicating. The first part of the manuscript is moving entirely toward capturing genuine communicative messages within ‘meaningless’ behavioral or verbal routines. For this purpose, he describes many sequences of autistic children observed at home during his Viennese medical profession. For example, Frankl reports the case of a child for whom the phrase ‘hello baby’—with which his father greeted him on his return at home before starting to play with him—had become his special verbal way to require anyone to play with him. Without the possibility of observing the autistic child in his natural environment, those words could have been considered only an echolalic pattern and not a way of communicating. In other words, Frankl has underlined that social interaction and communication is not absent, but qualitatively different in autistic people: they appear socially uninterested but actually they express their social interest in a less conformist way. This truly innovative idea in its day, remained hidden or controversial until recent years. For example, Jaswal and Akhtar [23] have presented an impressive array of evidence that individuals with autism retain a fundamental drive for social interaction and meaningful social relations.

### The idea of compensatory strategies

In the last part of his manuscript, Frankl hypothesizes that a pseudo-affective language can be developed as a compensatory strategy (when there is a lack of affective language) to cope with the human necessity of ‘being in contact with others’, which is not absent in autistic people. He furnishes four possible examples of alternative, ‘artificial’ languages:

1. The monotonous rote verbal production. According to Frankl, these vocal repetitions may assume a meaning and become a sort of substitute communicative system between the autistic subject and their primary caregiver;
2. The automaton-like language. Frankl observed that some children do not only talk like an automaton, but their whole body looks like a mere mechanic support: completely missing those gestures and corporeal attitude that are typical of human motor behavior;
3. The scanning language, that is a rhythmical language, yet lifeless and without emotional tone inflections. This is interpreted by Frankl as the effort to recapture, if not an affective language modulation, at least a modulated language structure;
4. The declamatory language, where feelings and emotions are reproduced in a very artificial manner, using an over-dramatized and exaggerated inflection of the voice, similar to what may be found in a theatrical performance.

These compensatory strategies are usually used by high-functioning autistic individuals, which are provided with sufficient, and sometimes out of the ordinary, cognitive abilities. The examples Frankl employed allows us to hypothesize that he wanted to claim for the possibility of teaching alternative languages to autistic children to further open communication between them and their caregivers. All of these pseudo-affective languages can be immediately perceived by the listener as something very different from a genuine expression of affect. They are example of the disconnection between affective (‘natural’ for Reid) and word (‘artificial’ for Reid) language, which involves difficulties in expressing themselves and their feeling. Again Thomas Reid wrote: ‘artificial signs signify, but they do not express; they speak to the intellect, but the affections hear them not’. If the integration of affective and word language is missing or compromised, the emotional engagement with others will also be disturbed and poor. In other words, in order to be in relation with others, people with autism try to compensate for their missing sense of affective language and its twin *affective contact,* the ability to form relations to others, not merely through a discursive understanding but also at a level of emotional attunement.

### Intersubjectivity

Anticipating the subject of intersubjectivity by many years, Frankl, in his manuscript, describes the relationship between a 10-month-old child and his mother. This contemporarily appropriate description of the social behavior of a 10-month-old, not yet talking baby, is reported by Frankl (pages 18–19) as an example for an isolated existence of the affective language before learning to talk. Already at this initial stage, Frankl describes a baby who is not only affectively bound to the other in a resonant, cyclic and dynamic relationship but also inextricably linked and influenced by the other’s corporeality—showcased by the fact that from birth, the baby is a body that expresses him/herself and is bound to the other’s embodied subjectivities in a reciprocal exchange. Frankl suggests that, in this case, instead of being informed by the child about what he is experiencing, one has to rely for this purpose on actions and on what it is supposed to be, non-communicative signs. Autistic people lack the spontaneous attunement that allows the subject to be in relationship with the other in an immediate manner without involving inferential or cognitive mechanisms. In other words, they lack affective (natural) language and, accordingly, affective contact, and they do not understand the affective language of others.

The notion of intersubjectivity [41], as well as of intercorporeality and interaffectivity [19], can be helpful to explore how ‘affective language’ and ‘affective contact’ can offer a new perspective on autism. In typical development, babies intuitively understand emotions (for example the rage) in others’ gestures or facial expressions. Without the intervention of simulations or inferential capabilities, babies can perceive the other’s corporeal movements as expressive and intentional and can immediately understand the other as a subjective agent and not as an object. Non-verbal affective language speaks directly to intersubjectivity. Thus, intercorporality becomes synonymous with intersubjectivity, and the language seems to be the medium of bodily, shared meanings. Corporeal gestures and linguistic praxis—as for Frankl, affective language and word language—form a coherent whole, and babbling and later on words seems to be an extension of the body as continuously and dynamically open towards the world. This kind of pre-reflective openness seems to be the very first form of intersubjectivity, which emerges from the implicit understanding of how initiatives for movement and action of the other are displayed. Accordingly, Trevarthen and Delafield-Butt [42] have proposed autism as a disorder principally of the primary affective and intentional quality of movement that underpins expressive communication and adaptive function, which thwarts development of social engagement and cognitive function.

## The Frankl’s modern view of autism

The arrival of George Frankl at Johns Hopkins with his Viennese concept of affective contact in his suitcase had set in motion a chain of events that inspired Kanner to develop his concept of autism, as was happening in Vienna for Asperger. Rediscovering the seminal role of Frankl in the early description of autism by Kanner and Asperger means we must also recognize his anticipation of the modern view of autism as distinct from the one that continued to influence the history of autism over the next 80 years.^5^

First, Frankl describes autism as a spectrum of conditions with variable degrees of severity. This inclusive account of autism, or “autistic condition” as Frankl called it in different part of the manuscript, has been denied and undervalued for many years. Recognizing that Asperger also observed marked individual differences in these individuals [15], we can hypothesize that the two clinicians, who worked together in Vienna, discussed the same cases and drew the same conclusion. In any case, this account is really modern. Even recently, as before the DSM-5 (2013) [1], autism was described as a state that included specific kinds of syndromes, such as those of Kanner and Asperger. This theory has turned out to be a very simplistic and dogmatic account, failing to record what Frankl had proposed as a condition with many nuances. For him, autism should be understood as part of a spectrum whose manifestations are not necessarily abnormal but expression of a developmental neurobiological condition that primarily needs to be understood by others.

Second, Frankl describes autism as a state of mind that is not necessarily abnormal. It is a *condition* and not a *disease*: what is at stake is the relationship between the subject and this condition and her/his power to cope with it or being trapped by it. If we take into account the historical time in which Frankl was living through, we can understand the importance and the need to emphasize that an autistic child was not a danger for the society but simply a person with a different ‘affective language’ (and a different ‘word language’) still able to communicate with others. Therefore, we claim that Frankl’s perspective was modern and innovative not only because he prioritized the role of the affective components, but also because he was developing inclusive therapeutic hypotheses starting from the diversity (neurodiversity, according to Baron-Cohen [5]) involved in communicating with the social world. What is crucial in Frankl’s account (and what still nowadays, after many years, must be taken into consideration) is the pain experienced by the autistic subject who struggles for the chance of being understood by others. According to Frankl, this is the real core of the autistic condition. The autistic child is not able to communicate like others because the affective language and the word language are not integrated into a coherent and harmonic whole. In this perspective, the impairment of the subject on the autistic spectrum does not affect primarily high-order cognitive functioning but more basic connections between affective and cognitive areas.

## The evolution of the concept of affective contact and of its neurobiological underpinnings

Frankl, in 1934 and 1943, emphasized the centrality of the “affective contact” as a dimension, whereas Kanner described the deficit of the affective contact simply as “autism”. This simple detail entails a huge difference between the two approaches. The word “autism” underlines the role of social detachment as the main deficit of the autistic state, the notion “affective contact” assumes the deficit in the emotional language as the real core of the disorder. We should remember that Kanner, in his paper dated 1943, did not include the deficit in non-verbal language among the diagnostic criteria for autism. He did not offer any examples of weakness in corporeal and gestural language, while Frankl considered the deficit in corporeal language the distinctive feature of the autistic condition.

George Frankl concludes his paper on *Nervous Child* in 1943 (the twin paper of Kanner’s famous one) with these words:

“We have become used to considering gestures a somewhat superfluous relic from the times when the ancestors of *Homo Sapiens*, in want of words and in need of some means of communication, used motor and vegetative-motor reactions in order to intimidate their enemies or to attract friends. This description reveals only the origins of gestural symbols as means of communication. But our clinical cases show that gestures are not merely a transitional remainder from olden times. It appears rather that the communication of emotions by gestural symbols is an important and well established function that is by no means destined to become extinct as long as emotions play an important role in human interactions.” [18, p. 262]

This seems like a precious legacy that will take a long time to be taken into consideration in the study of typical and atypical development of modern, technically complex, human social relations, or those primitive ones of *Homo Sapiens*, as Frankl says.

Perhaps the time was not right to investigate the pivotal role that motor gestures have as an essential part of an affective language, in which vocal prosody and visual expression are obviously also hugely important. This intuition of George Frankl on the primacy of the non-verbal over the verbal would have needed a new neuroscientific field. It is worthwhile here to signal that in those years the British neurologist MacDonald Critchley published a pioneering work ‘The Language of Gesture’ [8], speculating that gesture may be the precursor of speech. In the ‘60 s Stern [38] discovered, through microanalysis of movies of natural play, that mother and infant communicate feelings by exchanging gestures of hands and face expressions in mutually mediated ‘conversations’ by exchanges of actions. In 1999, Corballis postulated that language has a gestural origin, as it evolved from arm postures that were gradually integrated with mouth vocalization. In this framework, *Sapien* communication systems are thought to emerge phylogenetically and ontogenetically from the interaction of gesture and speech. Nowadays known as the human ‘mirror neuron system’ [11, 33], these interactive sensory-motor systems would constitute the shared neural substrate for both comprehension and production of cooperative action and language [32]. In particular, brain stimulation studies identified the Inferior Frontal Gyrus (IFG) as an important region of the mirror neuron system mediating the integration between gesture and speech [21, 45]. Crucially, the IFG has been consistently observed as a cortical region altered in neuroimaging studies of ASD individuals [6, 22, 31], thus potentially contributing to the disruption in ASD of the integration of gesture-speech, or ‘affective language’ and ‘word language’, according to Frankl. Another mirror mechanism for expression and recognition of affective action and language situated in the insula has been recently suggested by Di Cesare and colleagues [13] who also proposed that an incorrect functioning of the dorso-lateral part of the insula—or of cortical areas functionally connected with it such as the IFG—could be responsible for difficulties in mirror mechanisms of children with autism [12].

A new line of research into these disruptions in autism research has been developed from the notion of ‘vitality forms’, a term introduced by Stern [39] to describe ‘how’ (not ‘why’ or ‘what’) an action is performed. Vitality forms (a concept that is close to affective language for Frankl) are considered a fundamental element of social interaction [20]. Along those lines are the twin papers by Di Cesare on the implicit understanding of rude versus gentle actions [12] and on difficulties of children with ASD in their discrimination [13]. These papers consider the impairment in recognizing ‘vitality forms’ a core constituent of ASD that has roots in atypical processing of basic-level sensorimotor information. It is suggested that in ASD this atypical sensorimotor processing impairs information conveyed by body movements or faces that is affective language following the pioneering work of George Frankl.

## Conclusion

In his published and unpublished papers, Frankl had developed original ideas on weakness of affective language as a key to understanding autism. These ideas, which have remained hidden for a long time, have actually been developed through studies on the importance of gestures in neurology [8], in phylogenesis [7], in ontogenesis [38, 41] and finally through the definition of the neural bases of ‘vitality forms’ proposed by Stern [39]. In our opinion, this was what struck Kanner and probably also Asperger. In the history of autism, the role of Frankl cannot be relegated to that of a messenger dove through which Kanner knew Asperger’s work. In other words, Frankl was not the intermediary to inform Kanner of Asperger’s work in Vienna, as often suggested. He was rather the person whose imaginative reflections on children he had observed in Vienna, and in Baltimore, allowed both Asperger and Kanner to develop their own ideas about atypical children. Indeed, perhaps precisely because the source of the ideas that were forming in their mind was common, both used the term autism although in a different senses. No coincidence then.

## Footnotes

1 We started our inquiry in October 2018. It is interesting to notice that on September 2019 Samantha Dluzak uploaded on Research Gate a preprint paper that strengthens our focus on Frankl’s role.

2 Recently, Simmonds and Sukhareva [37] translated and published the second part of Sukhareva’s paper, where she describes five cases of girls, which seem to be on the autistic spectrum. This contribution not only shed lights on the role of female researchers in the history of autism, but it is also important to fill the existing gap in the literature about autism in girls and women.

3 The name of Asperger has been at the center of a wide discussion about his presumable link with Nazi persecution: while some authors like Czech [9, 10] and Sheffer [35] explicitly accused him of being actively involved in the euthanasia program that took place in the *Am Spiegelgrund,* Dean Falk [15] recognized only his passive and unaware presence in the Nazi program and, on the contrary, talked about an ‘individual resistance’.

4 However, it is possible that Frankl was aware of Asperger's work: in this regard, Dluzak [14] reported contacts between Frankl and Asperger around 1950.

5 We, Muratori and Bizzari, introduced these features in the paper ‘Autism as a disruption of affective contact: the forgotten role of George Frankl’ published in 2019 [29].
